# Dopamine Agonists and their risk to induce psychotic episodes in Parkinson's disease: a case-control study

**DOI:** 10.1186/1471-2377-9-23

**Published:** 2009-06-10

**Authors:** Daniel Ecker, Alexander Unrath, Jan Kassubek, Michael Sabolek

**Affiliations:** 1 Department of Neurology, University of Ulm, 89081 Ulm, Germany; 2 Current address: Department of Neurology, EMA-University of Greifswald, 17475 Greifswald, Germany; 3 Current address: Focus Clinical Drug Development, Neuss, Germany

## Abstract

**Background:**

Psychosis is rare in untreated patients with Parkinson's disease (PD) but the prevalence rises to 40% during dopaminergic treatment. So far, no systematic comparison of the psychogenic potential of different dopaminergic drugs had been performed.

**Methods:**

Eighty PD patients with psychotic episodes were compared to an age-matched control group of PD patients without psychotic episodes (n = 120) in a cross-sectional retrospective study.

**Results:**

We found a positive correlation between psychotic episodes and dementia, number of concomitant medication, and pergolide intake. Odds ratio calculation confirmed the association with dementia. With respect to dopaminergic treatment, pergolide showed the highest odds ratio, levodopa the lowest. An adjusted logistic regression model confirmed the strong association with psychotic episodes and pergolide and no association with levodopa (adjusted odds ratio 2.01 and 0.11, respectively).

**Conclusion:**

The analysis indicates that dementia and concomitant medication are factors in PD associated with psychotic symptoms. Furthermore, different dopaminergic drugs showed markedly different associations with psychotic symptoms

## Background

Idiopathic Parkinson's disease (PD) is the second most common neurodegenerative disorder. Although it is mainly classified as a movement disorder, non-motor symptoms such as psychiatric complications are a regular phenomenon during disease progression [1,2]. Although less common than depression [3], psychosis is well-known as a highly relevant psychiatric symptom in PD. Psychotic symptoms occur only in a minority (less than 10%) of untreated PD patients but are known to be associated with dopaminergic treatment [4-6]. Hallucinations or illusions are observed in up to 40% of PD patients treated with dopaminergic drugs [7-12]. Psychosis in PD patients is associated with excessive disability, worse quality of life, poor outcome, caregiver distress [1,2], cognitive impairment, and sleep disturbances [10]. There are multiple factors which are considered to elevate the risk for the development of psychotic syndromes, i.e. higher age, duration of disease, microvascular changes, brain atrophy, polypharmacotherapy, cognitive impairment, depression, and sleep disturbances [7,8,10,12-15]. In addition, the application of anticholinergic drugs, other reasons for a cholinergic deficit or temporal Lewy bodies can contribute to the development of psychosis in PD patients. In patients with hypokinetic movement disorders distinct from PD, such as Dementia with Lewy bodies (DLB) or Progressive Supranunclear Palsy (PSP), psychosis is also a common phenomenon [16-18], but this has not been addressed in this study.

Dopaminergic treatment seems to be a particular risk factor for psychoses in PD. Several reports have described an induction of psychosis by most of the common dopamine agonists (DA) [19-26]. Papapetropoulos et al. concluded that virtually all anti-Parkinsonian drugs are able to induce psychotic symptoms [27]. The question whether certain dopaminergic drugs do have a higher potential to induce psychosis than others is discussed in a controversial manner. In a study of Aarsland et al., the authors found no correlation at all between anti-Parkinsonian drugs (levodopa and DA) and hallucinations [12]. In contrast, a prospective comparison of patients treated with either levodopa or ropinirole demonstrated a higher incidence of hallucinations in the ropinirole group (17% vs. 6%) [24,28]. In the present retrospective study, possible correlations between dopaminergic treatment with different drugs and the risk for PD patients to develop psychosis were analyzed.

## Methods

### Kind of study

Monocentric, retrospective, cross sectional case control study. The study was approved by the ethics committee of the University of Ulm, Germany (reference number: EK 18/09).

### Patient sample

The archives of the outpatient clinic for Movement Disorders, University of Ulm, Germany were screened for patients with PD who simultaneously had a history of psychotic symptoms (hallucinations, illusions, delusions, paranoia) in the observation period between 2000 and 2005 as documented in the patient charts. In total, 1,200 patient charts were reviewed. The chart review was performed by trained neurologists with long standing experience in movement disorders. Only patients with a PD according to the "UK brain bank criteria" were included, and only patients with dopaminergic medication in approved dosages were included. No off-label-use was allowed. Patients with anticholinergic drugs were not considered for the analysis. Cholinesterase inhibitors were not yet approved for the treatment of dementia in PD in the observation period and were therefore not used. Amantadine, which is known to be able to induce psychotic symptoms, was even less frequent in the group with psychosis than in the control group (psychosis group, 1.25% vs. controls, 12.50%). In addition, patients with other tentative aetiology that may be responsible for psychosis such as infections were also excluded, as well as patients with incomplete or vague data. For n = 80 patients, we found a complete dataset. This sample was compared to an age-matched control sample (n = 120) of PD patients without any recorded psychotic symptoms in the same observation period.

### Investigated Parameters

In all patients who were included in the analysis, psychosis had been diagnosed during the treatment in the outpatient clinic. The occurrence of psychotic symptoms was collected according to the questions of the "Neuropsychiatric Inventory" [29]. The occurrence of psychotic symptoms was correlated with the dopaminergic drug treatment at the time of diagnosis of psychotic symptoms, patient age, all concomitant medication and all concomitant diseases as documented in the patient chart, white matter lesions and cortical brain atrophy as described in the MRI results in the patient chart, and dementia. The clinical-radiological evaluation as recorded in the patient chart was used, no re-evaluation of the MRI scans was performed. Dementia was diagnosed by trained neuropsychologists with a long standing experience in dementia in PD on the basis of a neuropsychological screening according to the "Screening for Cognitive Impairment" proposed by Beinhoff et al. [30] after an initial Mini Mental Status Examination performed by the neurologist. This screening method included the Letter Sorting Test (LST) for the assessment of working memory and concentration, the subscale orientation of the ADAScog for assessment of orientation, the Memory Impairment Screen (MIS) for assessment of memory, the Boston Naming Test (BNT) for assessment of naming deficits, a test of verbal fluency (VF), and the clock drawing test (CDT). Values of 5 or less in the MIS and 20 or less in VF, respectively, were proposed by the authors as thresholds to indicate for mild cognitive impairment and dementia. We limited the analysis to the most frequently used dopaminergic drugs in this sample (pergolide, cabergoline, ropinirole, pramipexole, and levodopa), since the number of patients treated with other DAs were too low for statistical analysis. All investigated dopaminergic drugs were used with maximal dosages as follows, levodopa 1000 mg/d, pergolide, 6 mg/d, cabergoline 6 mg/d, ropinirole 24 mg/d, pramipexole 2.8 mg/d. The investigated dopaminergic drugs were compared with regard to their action at D-, 5-HT- and α_2_-receptors.

### Statistics

SAS software (SAS institute, Cary, NC) was used in a first step for correlation analysis (Spearman-Pearson) and the calculation of odds ratios. In a second step, the data were analyzed using a logistic regression model adjusted for sex, dementia, concomitant medication, concomitant disorders and dosages (levodopa equivalence doses). For the calculation of the levodopa equivalence doses we used the following values, levodopa × 1; levodopa-CR × 0,75; pramipexole × 67; ropinirole × 16,67; pergolide × 100; bromocriptine × 10, cabergoline × 100 which orientate at the official guidelines for diagnostic and therapy of Parkinsonian syndromes of the German neurological society (DGN), 4th revised version 2008. MRI-based parameters (white matter lesions and cortical brain atrophy) could not be included in this model since only a limited number of patients (n = 84 in the group with psychotic symptoms, n = 50 in the control group) had a high quality MRI scan. The disease duration, which may also have an influence on the probability to develop psychotic symptoms, was not included in the analysis, since not all patients treated in our outpatient clinic were also initially diagnosed in our clinic. Therefore, only records with unconfirmed history data were available which we did not use. P values < 0.05 were considered statistically significant.

## Results

### Demographic data

Between the group of PD patients with a positive history of psychotic symptoms (n = 80, mean age 72 years, 54% male, 46% female) and the control group (n = 120, mean age 72 years, 69% male, 31% female), there were no significant differences in age, gender and number of concomitant diseases (Table 1). In the group with psychotic symptoms 21.25% took cabergoline, 10.00% took pramipexole, 21.25% took pergolide and 23.75% took ropinirole. In the control group, 25.83% took cabergoline, 17.50% took pramipexole, 10.83% took pergolide and 20.83% took ropinirole.

**Table 1:** Overview over epidemiological date of the sample of psychotic and non-psychotic patients

**Parameters**	**Psychosis**	**No Psychosis**
**N**	80	120
**Age**	72.2 (± 8.6)	71.9 (± 7.1)
**sex ratio f/m**	0.82	0.75
**concomitant diseases**	3.1 (± 2.1)	3.7 (± 2.3)

Mean values of parameters with standard deviation in brackets. N = number of patients, f = female, m = male

### Correlation analysis

The highest positive correlation was found for the occurrence of psychotic episodes and dementia (p = 0.007), followed by the number of concomitant diseases (p = 0.020), the number of concomitant medication (p = 0.039), and pergolide intake (p = 0.044). There was a trend for the correlation of psychotic symptoms and cortical brain atrophy (p = 0.072) as well as for the presence of white matter lesions (p = 0.33). There was no significant correlation between the occurrence of psychotic symptoms and the use of other dopaminergic medication (ropinirole, p = 0.731; pramipexole, p = 0.103; cabergoline, p = 0.171). In contrast, there was a significant correlation between fewer psychotic episodes and levodopa treatment (p < 0.001).

### Odds ratio

Odds ratio calculation showed the highest risk for psychotic episodes in patients with cortical brain atrophy and dementia. With respect to dopaminergic treatment, pergolide showed by far the highest odds ratio, whereas levodopa treatment showed the lowest one; for a synopsis of odds ratios and confidence intervals, cf. Table 2.

**Table 2:** Overview of odds ratio calculation and logistic regression model data.

	**Odds ratio**	**Adjusted Odds**
**cortical atrophy^1^**	2.47 (1.83–18.62)^1^	NA
**dementia**	1.98 (1.03–5.51)	NA
**white matter lesions^1^**	1.19 (0.54–5.51)^1^	NA
**pergolide**	**2.22 **(1.01–4.87)	**2.01 **(1.22–5.45)
**ropinirole**	1.18 (0.60–2.32)	1.05 (0.55–2.11)
**pramipexole**	0.52 (0.22–1.24)	0.94 (0.33–1.66)
**cabergoline**	0,32 (0.16–0.63)	0.65 (0.39–1.09)
**levodopa**	**0.14 **(0.07–0.26)	**0.11 **(0.06–0.19)

^1^Calculated for n = 89. Confidence interval in brackets. Pergolide and levodopa data in bold for accentuation. NA = not applicable

### Logistic regression model

**Figure 1:**
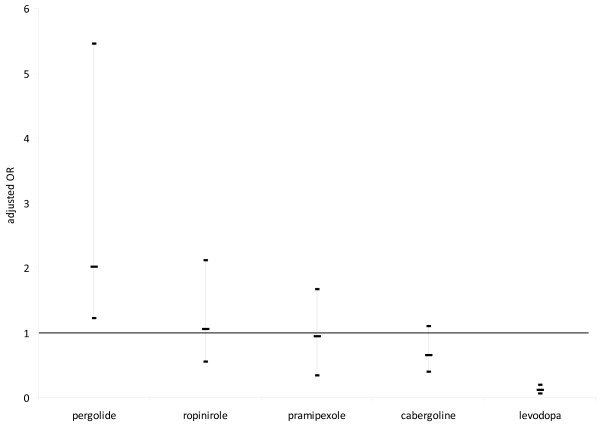
**Adjusted odds ratios for levodopa, cabergoline, pramipexole ropinirole and pergolide**. Results of the logistical regression model adjusted for sex, dementia, concomitant medication and concomitant disorders. Box marks indicate odds ratio, vertical bars indicate confidence intervals. Horizontal line indicates odds ratio of 1.

The logistic regression model (Table 2/Figure 1) confirmed these results. Pergolide had an adjusted odds ratio of 2.01 showing a markedly increased risk, and levodopa had an adjusted odds ratio of 0.11 showing a decreased risk for the occurrence of psychotic symptoms. For all other dopaminergic drugs investigated, statistically reliable statements were not possible due to adjusted odds near 1 and overlapping confidence intervals (Figure 1).

## Discussion

The occurrence of psychotic episodes in PD patients was shown to correlate with the presence of dementia and the numbers of concomitant diseases and of concomitant drugs, whereas MRI-based factors of cortical atrophy and white matter lesions could not be finally assessed due to limited data. With respect to dopaminergic treatment, pergolide was associated with a significantly increased risk for the development of psychotic symptoms, whereas levodopa was found to bear the lowest risk of all investigated dopaminergic drugs. In the literature, there is a large variability in the reported incidence of PD-associated psychosis among different authors, particularly depending on the method used [7-12,31]. Prospective studies mostly show higher incidence rates than retrospective investigations. In one retrospective study with outpatients, the incidence was as low as 3% [31]. Some investigators found no clear association between psychosis in PD and dopaminergic treatment itself [32], but reported a correlation between psychosis in PD and polypharmacotherapy [33]. In the present study, a positive correlation between the number of overall used drugs per patient and the risk to develop psychotic symptoms was observed.

Aarsland et al. described a correlation between higher age and the incidence of psychosis in PD patients [12]. The peak age of onset of PD is 55 to 66 years [34], while the median age of our PD patient sample with psychotic episodes was 72 ± 9 years. Whether this indicates a correlation of psychotic symptoms with age itself or with the duration of the disease, as described previously by Fenelon et al. [9] cannot be distinguished as no reliable data concerning disease duration are available in all patients (see "Statistics")

Based upon the consideration that a main risk factor for the development of psychosis in PD is the application of dopaminergic medication itself [5,9,12], it was our aim to investigate the potential of different dopaminergic drugs to induce psychotic episodes. In randomized double-blind trials investigating different DAs the incidence of hallucinations was 8.1% for priribedil, 2.8% for bromocriptine, 4% for rotigotine, 5% for ropinirole, 7%–9.3% for pramipexole, 4.8% cabergoline, 3.4 for pergolide, 0% – 4.4% for levodopa [35-40]. As in these studies often early PD stages (Hoehn and Yahr stage 1–2.5) without severe concomitant diseases are enrolled, these values represent not necessarily the situation in "real life".

This study was performed in the assumption that each included PD patient was treated with an individually optimized DA dose with respect to the efficacy on motor symptoms. By far the highest association for the induction of psychotic episodes was observed for pergolide. Since this association was much lower for cabergoline, i.e. also an ergoline DA, the high risk for psychosis in pergolide treatment cannot solely be explained as a group effect.

All investigated DAs behave as highly effective D_2 _agonists [41] and, to a lower extent, D_3 _agonists [42]. Therefore actions at other receptors, as for example agonism at D_4 _receptors may play a role. Remarkably many antipsychotic agents show marked antagonism at D_4 _receptors [43]. Levodopa – the drug with the lowest risk for psychosis in the present investigation – has only low affinity to D_4 _receptors [43]. Also cabergoline and pramipexole, the two DAs which showed the lowest adjusted odds ratios in this study (0.58 and 0.64, respectively) have a lower agonistic efficacy (E_max _49 and 42) at the D_4 _receptor [41] than pergolide (E_max _56, adjusted odds ratio 2.8). Another noticeable point is the investigated DA's α_2 _receptor affinity: Only pergolide shows a significant agonistic effect at α_2 _receptors. In contrast, cabergoline behaves as a strong antagonist at α_2 _receptors, whereas ropinirole and pramipexole are mostly inactive at α receptors [41]. Further, both cabergoline and pergolide are 5-HT_2A-C _receptor agonists [44] and especially 5-HT_2A _receptors are suspect to be related to the development of hallucinations [45,46]. In the comparison of all hereby investigated DAs, pergolide shows the strongest agonistic effect at the 5-HT_2A _receptor [44]. This profile of pergolide as a strong agonist at 5-HT_2 _and α_2 _receptors resembles the pharmacological profile of amphetamine derivates [45]. Therefore we conclude that the unique pharmacological profile of pergolide (strong D_2 _and D_4 _receptor agonism in combination with strong 5-HT_2a _and α_2 _receptor agonism) might contribute to the high association of pergolide with the induction of psychosis.

Our analysis confirmed that levodopa shows the lowest risk of all investigated drugs for the development of psychotic episodes in PD. In accordance with this result, Holroyd et al. found no association between dose and duration of levodopa treatment and the development of hallucinations at all [32]. Thus, the common clinical practice to use levodopa in psychotic PD patients rather than DAs is plausible.

Because of the design as a monocentric, retrospective, cross sectional study, there are certainly intrinsic limitations to the results. We are aware that the influence of different dopaminergic drugs is difficult to interpret. The dosages are hard to compare due to different pharmacokinetic and pharmacodynamic characteristics of the different DA. There is also no generally accepted "levodopa equivalent dose". The levodopa equivalent doses of different dopaminergic drugs vary substantially since they result in general from the consensus of an expert committee and refer only to the effect on the motor symptoms. Nevertheless we included the calculated levodopa equivalence doses as a confounder to the logistic regression model to exclude significantly higher dosage in the psychosis group.

## Conclusion

Being well aware of the intrinsic methodological limitations which this retrospective, cross sectional study has, we could nevertheless demonstrate by systematic data analysis in a well defined patient sample that PD-associated psychosis correlates both with epidemiological factors and with the choice of different dopaminergic drugs. These factors might be included in differential therapeutic considerations in order to minimize the risk of psychosis.

## Competing interests

The authors declare that they have no competing interests.

## Authors' contributions

DE, AU and MS performed the chart review and the data collection. DE performed the statistical analysis. DE, AU, JK and MS performed the discussion of the manuscript ED drafted the manuscript, JK and MS finalized the manuscript. All authors read and approved the manuscript.

## Pre-publication history

The pre-publication history for this paper can be accessed here:


